# Effects of sex and deletion of neuropeptide Y2 receptors from GABAergic neurons on affective and alcohol drinking behaviors in mice

**DOI:** 10.3389/fnint.2013.00100

**Published:** 2013-12-25

**Authors:** Nora M. McCall, Gretchen M. Sprow, Eric Delpire, Todd E. Thiele, Thomas L. Kash, Kristen E. Pleil

**Affiliations:** ^1^Bowles Center for Alcohol Studies, University of North Carolina School of MedicineChapel Hill, NC, USA; ^2^Department of Pharmacology, University of North Carolina School of MedicineChapel Hill, NC, USA; ^3^Department of Psychology, University of North Carolina at Chapel HillChapel Hill, NC, USA; ^4^Department of Anesthesiology, Vanderbilt UniversityNashville, TN, USA; ^5^Department of Molecular Physiology and Biophysics, Vanderbilt UniversityNashville, TN, USA

**Keywords:** NPY, anxiety, depression, ethanol, amygdala, female, GABA

## Abstract

A large literature has demonstrated that neuropeptide Y (NPY) regulates many emotional and reward-related behaviors via its primary receptors, Y1R and Y2R. Classically, NPY actions at postsynaptic Y1R decrease anxiety, depression, and alcohol drinking, while its actions at presynaptic Y2R produce the opposite behavioral phenotypes. However, emerging evidence suggests that activation of Y2R can also produce anxiolysis in a brain region and neurotransmitter system-dependent fashion. Further, numerous human and rodent studies have reported that females display higher levels of anxiety, depression, and alcohol drinking. In this study, we evaluated sex differences and the role of Y2R on GABAergic transmission in these behaviors using a novel transgenic mouse that lacks Y2R specifically in VGAT-expressing neurons (VGAT-Y2R knockout). First, we confirmed our genetic manipulation by demonstrating that Y2R protein expression was decreased and that a Y2R agonist could not alter GABAergic transmission in the extended amygdala, a limbic brain region critically implicated in the regulation of anxiety and alcohol drinking behaviors, using immunofluorescence and slice electrophysiology. Then, we tested male and female VGAT-Y2R knockout mice on a series of behavioral assays for anxiety, depression, fear, anhedonia, and alcohol drinking. We found that females displayed greater basal anxiety, higher levels of ethanol consumption, and faster fear conditioning than males, and that knockout mice exhibited enhanced depressive-like behavior in the forced swim test. Together, these results confirm previous studies that demonstrate higher expression of negative affective and alcohol drinking behaviors in females than males, and they highlight the importance of Y2R function in GABAergic systems in the expression of depressive-like behavior.

## INTRODUCTION

Neuropeptide Y (NPY) is an endogenous “anti-stress” neuropeptide involved in the regulation of several affective and reward-related behaviors. Genetic and pharmacological manipulations of central NPY have shown that this peptide plays an important role in anxiety, depression, and alcohol drinking behavior (Heilig et al., 1989; Bannon et al., 2000; Lindell et al., 2010; Tasan et al., 2010; Sparrow et al., 2012). NPY’s primary receptors, Y1R and Y2R, are highly expressed throughout the brain (Parker and Herzog, 1999) and NPY signaling via these two G protein-coupled receptors are thought to produce diverging behavioral phenotypes. While Y1R has generally been shown to mediate many of the anxiolytic and anti-drinking effects of NPY (Schroeder et al., 2003; Karlsson et al., 2008; Bertocchi et al., 2011; Sparrow et al., 2012), Y2R has been shown to increase anxiety and alcohol consumption (Tschenett et al., 2003; Bacchi et al., 2006; Tasan et al., 2010; Sparrow et al., 2012). However, there are several reports indicating that NPY’s Y2R-mediated behavioral effects are much more complex (Kask et al., 1998; Thiele et al., 2004; Fendt et al., 2009; Zambello et al., 2010; Trent and Menard, 2013). For example, Y2R mediates the anxiolytic effects of NPY in the lateral septum and locus coeruleus (Kask et al., 1998; Trent and Menard, 2013).

Several lines of evidence suggest that the varying behavioral outcomes of Y2R manipulations may depend on the neurotransmitter systems that Y2R modulate in these regions. Y2R has been described as having an inhibitory role in the release of several neurotransmitters, including glutamate, GABA, norepinephrine, and dopamine, among others (Silva et al., 2005; Zambello et al., 2010). Presynaptic Y2R decreases glutamate release in the hippocampus and decreases GABA release in the central nucleus of the amygdala (CeA), and the bed nucleus of the stria terminalis (BNST; Kash and Winder, 2006; Gilpin et al., 2011; Ledri et al., 2011; Pleil et al., 2012), extended amygdala structures particularly implicated in a host of anxiety, depression, and alcohol drinking behaviors (Schroeder et al., 2003; Tasan et al., 2010; Zhang et al., 2010; Gilpin et al., 2011). For example, we have recently shown that stress alters Y2R-mediated modulation of GABAergic transmission in the BNST of stress-susceptible DBA/2J, but not stress-resilient C57BL/6J, mice (Mozhui et al., 2010; Pleil et al., 2012). In the present study, we evaluated the role of Y2R modulation of GABAergic transmission in anxiety, depression, and alcohol drinking behaviors using a transgenic mouse lacking Y2R in GABAergic neurons.

A robust human literature has shown that females display greater propensity for these affective and drinking behaviors than males (Kessler et al., 1994, 1995; Gater et al., 1998; Lewinsohn et al., 1998a,b; Bekker and van Mens-Verhulst, 2007; Ohrmann et al., 2010). While many rodent studies reveal similar results, particularly in mice on a C57BL/6J background (Kennett et al., 1986; Johnston and File, 1991; Middaugh et al., 1999; Frye et al., 2000; Adamec et al., 2006; Dalla and Shors, 2009; Stack et al., 2010; ter Horst et al., 2012b; Melon et al., 2013), a number of studies report that male rodents exhibit greater anxiety-related behavior, depending on the strain and behavioral assay used (Johnston and File, 1991; Rodgers and Cole, 1993; Zimmerberg and Farley, 1993; Leret et al., 1994; Voikar et al., 2001; Adamec et al., 2006; ter Horst et al., 2012a). Therefore, we used both male and female mice to investigate potential sex differences in these behaviors and the role of GABAergic Y2R.

## MATERIALS AND METHODS

### SUBJECTS

We generated mice that lack Y2R specifically in GABAergic neurons (VGAT-Y2R knockout) by breeding heterozygous VGAT-ires-Cre mice (Vong et al., 2011) with homozygous Y2R-floxed mice. Y2R-floxed mice were generated using the targeting protocol described in the following section. Female offspring heterozygous for Y2R-flox and vGAT-ires-Cre were bred with male homozygous Y2R-floxed mice. Homozygous Y2R-floxed offspring from this breeding scheme were used in this study; VGAT-ires-Cre +/-, Y2R-floxed +/+ mice had Y2R knocked out from VGAT neurons (VGAT-Y2R KO) and VGAT-ires-Cre -/-, Y2R-floxed +/+ mice did not have altered Y2R expression (VGAT-Y2R control). Mice were group-housed in our colony room with a 12:12-h light–dark cycle with lights on at 7 a.m. Mice had *ad libitum* access to standard rodent chow and water. All procedures were approved by the Institutional Animal Care and Use Committee of the University of North Carolina at Chapel Hill and performed in accordance with the National Institutes of Health guide for the care and use of laboratory animals. Mice were at least 6 weeks of age at the beginning of the study.

### *NPY Y2R* TARGETING

**FIGURE 1:**
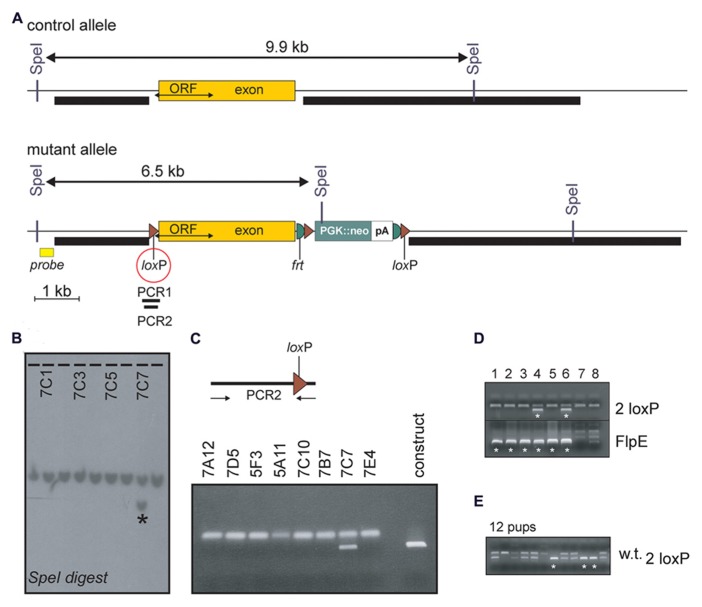
**Disruption of mouse *NPY Y2R*. (A)** Structure of the *NPY Y2R* control and mutant alleles around exon 2. The entire Open Reading frame (ORF) of the receptor is contained within the exon. Position of the 5′ probe, of the short and long arms of recombination (thick black bars), of loxP and FRT sites, and of *Spe*I restriction sites are indicated. The first loxP site, which is at risk of not being included during recombination, is highlighted by a red circle. Presence of this loxP site is verified by PCR amplification of ES cell genomic DNA (PCR1 and PCR2). **(B)** Portion of the Southern blot analysis of ES-cell genomic DNA digested with *Spe*I and probed with the 5′ probe. Signals are from clones 7B12 to 7C8. The wild-type gene shows across as a band of ~10 kb whereas a 6.5 kb additional band is observed in clone 7C7. **(C)** Pre-sence of the loxP site is confirmed in ES cell clone 7C7. The construct serves as positive control. **(D)** Elimination of the neomycin resistance gene cassette is illustrated in two mice by the presence of the *Flp*E transgene (stars) and the presence of a larger band in samples 4 and 6 (also indicated by stars). **(E)** PCR genotyping of 21-day old pups demonstrating that viable Y2R-floxed mice were successfully generated. PCR yielded two samples with a single small band (wild-type mice), seven samples with two bands (heterozygous two loxP mice) and three samples with a single large band (stars, homozygous two loxP mice).

Using recombineering, a 2.1 kb genomic fragment was dropped from BAC clone bMQ-343H17 (Geneservice, Ltd, UK) upstream of a loxP site located in modified pBSK+ vector. This fragment constitutes the left arm of recombination (**Figure 1A**). Similarly, a 6.3 kb fragment was dropped from the same BAC clone downstream of a loxP site located in a separate pBSK+ vector containing a neomycin-resistance gene cassette flanked by two FRT and loxP sites. This fragment contains the right arm of recombination. Finally, a 3.1 kb fragment consisting of exon 2 was dropped to a third pBSK+ vector. The targeting vector was then created by adding the fragment containing the left arm of recombination and the fragment containing the exon to the vector containing the neomycin resistance gene cassette and the right arm of recombination using unique restrictions sites. The construct was linearized with *Not*I and electroporated in TL-1 embryonic stem (ES) cells. The cells were the plated and grown on fibroblast feeder cells in Dulbecco’s modified Eagle medium (DMEM) supplemented with 15% fetal bovine serum [Life Technologies (formerly Invitrogen), Carlsbad, CA, USA], 50 μg ml^-^^1^ gentamycin (Invitrogen), 1,000 U ml–1 murine leukemia inhibitory factor [Millipore Biosciences (formerly Chemicon), Temecula, CA, USA], 90 μM 2-mercaptoethanol (Sigma-Aldrich, St. Louis, MO, USA) and 0.2 mg ml^-^^1^ G418 (Invitrogen). We picked 494 independent neomycin-resistant colonies and grew them in 96-well plates on a feeder layer, and then expanded the colonies and analyzed them for the presence of the mutated gene. Southern-blot analysis was done using genomic DNA digested with *SpeI*I and hybridized with a ^32^P-labeled probe consisting of a 409-bp PCR fragment located upstream of the left arm of recombination (**Figure 1A**). Eight clones contained a 6.5-kb *Spe*I fragment characteristic of the mutated gene (**Figure 1B**). After further characterization using internal and 5′-end probes, PCR amplification ES cell genomic DNA was then used to assess the presence of the first loxP site. Indeed, as the targeted exon is large, it is likely to participate in the recombination in lieu of the left arm resulting in failure to incorporate the loxP site. In fact, only one clone demonstrated the presence of the loxP site (**Figure 1C**). This properly recombined ES cell clone was injected into C57BL/6J blastocysts and three chimeric mice with ~90% brown fur were generated. After confirmation of germline transmission, mice carrying the three loxP allele were mated with *Flp*E mice to eliminate the neomycin-resistance gene cassette. As seen in **Figure 1D**, mice positive for the *Flp*E transgene demonstrated presence of the two loxP allele. Homozygous Y2R-floxed mice were then successfully generated by mating heterozygous two loxP mice (**Figure 1E**). Out of six breeding cages, 55 pups were generated, including 15 homozygotes, 10 controls, and 30 heterozygotes. The distribution was Mendelian as it corresponded to 27 (~25%), 18 (~25%), and 55% (~50%), respectively. To ensure that the exon could be excised by CRE recombination, females carrying one copy of the two loxP allele were crossed to E2a-CRE mice. Out of 17 pups produced, about ½ carried the E2a-CRE transgene and three demonstrated loss of the exon (data not shown).

### FUNCTIONAL AND ANATOMICAL CONFIRMATION OF GENETIC MANIPULATION

#### Slice electrophysiology

To examine the effects of NPY on evoked GABAergic transmission in the BNST, we performed whole-cell voltage-clamp electrophysiological recordings in dorsal BNST neurons from acutely prepared coronal brain slices, as previously described (Li et al., 2012; Pleil et al., 2012), of VGAT-Y2R mice. Briefly, mice were decapitated under isoflurane anesthesia, and their brains were rapidly removed and placed in ice-cold sucrose-artificial cerebrospinal fluid (ACSF) containing: (in mM) 194 sucrose, 20 NaCl, 4.4 KCl, 2 CaCl_2_, 1 MgCl_2_, 1.2 NaH_2_PO_4_, 10.0 glucose, and 26.0 NaHCO_3_ saturated with 95% O_2_/5% CO_2_. Coronal slices 300 μm in thickness containing the dorsal BNST (Bregma 0.26–0.02 mm) were sectioned on a Leica VT1200 vibratome and stored in a holding chamber with 28–30°C, oxygenated ACSF containing [(in mM) 124 NaCl, 4.4 KCl, 2 CaCl_2_, 1.2 MgSO_4_, 1 NaH_2_PO_4_, 10.0 glucose, and 26.0 NaHCO_3_]. Slices were transferred to a submerged recording chamber (Warner Instruments, Hamden, CT, USA), where they were perfused with heated, oxygenated ACSF (28–30°C) at a rate of approximately 2 ml/min and allowed to equilibrate for 30 min before electrophysiological recordings.

Recording electrodes (3–5 MΩ) were pulled with a Flaming–Brown Micropipette Puller (Sutter Instruments, Novato, CA, USA) using thin-walled borosilicate glass capillaries. Electrodes were filled with (in mM) 70 KCl, 65 K^+^-gluconate, 5 NaCl, 10 HEPES, 0.6 EGTA, 4 ATP, 0.4 GTP, pH 7.2, 290–295 mOsmol. To block postsynaptic sodium spikes, lidocaine *N*-ethyl bromide (1 mg/ml) was included in the intracellular recording solution. Neurons were held at -70 mV and electrically evoked GABA_A_ receptor (GABA_A_R)-mediated inhibitory postsynaptic currents (eIPSCs) were pharmacologically isolated during recordings by adding 3 mM kynurenic acid (Abcam, Cambridge, UK) to the bath solution to block AMPA and NMDA receptor-dependent postsynaptic currents.

Twisted nichrome wire stimulating electrodes were placed dorsal to the recording electrode, 100–500 μm medial from the recorded neuron. eIPSCs were evoked at 0.1 Hz by local fiber stimulation with bipolar electrodes (5–50 V with a 100–150 μs duration) once every 10 s. Signals were acquired via a Multiclamp 700B amplifier and analyzed using Clampfit 10.3 software (Molecular Devices, Sunnyvale, CA, USA). After at least 5 min in which mean peak eIPSC amplitude was stable (“baseline period”), NPY (Abcam, Cambridge, UK), stocked in distilled water was diluted to 300 nM in the bath solution and applied to the bath for 10 min, followed by a washout period of at least 5 min. This concentration of NPY has previously been demonstrated to be maximally effective in slices from naïve mice in our lab and other labs (Kash and Winder, 2006; Chee et al., 2010; Pleil et al., 2012). Experiments were analyzed by measuring the peak amplitude of the synaptic response, which was normalized to the baseline period. Input resistance and access resistance were continuously monitored during all experiments, and those in which changes in access resistance were greater than 20% were not included in the data analysis.

#### Fluorescence immunohistochemistry

Mice were anesthetized with Avertin and perfused intracardially with 0.01 M phosphate buffer saline (PBS) followed by 4% paraformaldehyde (PFA) in PBS. Brains were extracted and post-fixed for 24 h in 4% PFA, then rinsed twice with PBS and immersed in 30% sucrose until saturated. Brains were hemisected and coronal slices 45 μm in thickness containing the BNST and CeA were prepared using a Leica VT1200 vibratome (Leica Microsystems, Nussloch, Germany).

All steps were performed at room temperature unless stated otherwise, using protocols included in the TSA amplification kit (Perkin Elmer, Waltham, MA, USA). Slices containing BNST and CeA were incubated in primary solution containing 0.3% Triton X-100, 0.5% bovine serum albumin, and an anti-Y2R antibody (1:3000; Neuromics, Edina, MN, USA) for 24 h at 4°C. Slices were washed in TNT buffer solution containing Tris/HCl, NaCl, and Tween20 for 10 min followed by TNB blocking solution containing TNT buffer with 0.5% blocking reagent provided in the TSA kit for 30 min. Slices were washed in TNB solution containing horseradish peroxidase (1:200) for 30 min and rinsed in TNT buffer. Sections were incubated in Cy3 (1:50) in amplification diluents provided in the TSA kit for 10 min and then rinsed in TNT buffer. All slices were mounted onto glass slides, allowed to dry, coverslipped with VectaShield (Vector Laboratories, Burlingame, CA, USA), and stored in the dark at 4°C.

Images of Y2R-IR in the BNST and CeA were obtained with an Olympus FV1000 inverted confocal microscope with a 10× objective and Olympus FluoView software. All serial sections throughout the BNST and CeA were used for quantification of Y2R-IR using ImageJ Software (National Institute of Health, Bethesda, MD). A contour was drawn around the dorsal BNST or CeA section to be analyzed; intensity values for all serial sections for each mouse were averaged to obtain one value per mouse for the BNST and CeA.

### BEHAVIOR

Behavioral assays for anxiety-like and depressive-like behaviors, as well as behavioral reactivity, were conducted at the UNC Mouse Behavioral Phenotyping Core during the lights-on phase of the day. Binge-like ethanol drinking was conducted in the home cage during the lights-off phase of the day using the standard Drinking-in-the-Dark (DID) paradigm, and ethanol and sucrose preference tests were conducted in the home cage across both phases of the light cycle.

#### Elevated plus maze

We used a standard elevated plus maze to assess anxiety-like behavior. Mice were placed in the center section facing an open arm and were allowed to freely explore the maze for 5 min. The number of entries into and time spent in open and closed arms of the apparatus was hand-scored by the experimenter.

#### Open field test

The open field test was used to assess general locomotor activity and anxiety-like behavior. Mice were placed in the corner of a 40 cm × 40 cm × 30 cm open field box (Versamax system, AccuScan Instruments) and allowed to freely explore the arena for 30 min. Behavioral testing boxes were contained inside sound-attenuating boxes with house lights and fans. Activity and position were tracked using beam breaks in the AccuScan Fusion Activity System and used to calculate distance traveled and time spent in the center of the field.

#### Light/dark box

The light/dark box was used to assess anxiety-like behavior. Mice were placed in the dark side of a 40 cm × 40 cm × 30 cm box containing light–dark box inserts (Versamax system, AccuScan Instruments) and allowed to freely explore the chamber for 15 min. Behavioral testing boxes were contained inside sound-attenuating boxes with houselights and fans. Activity and location were tracked using beam breaks in the AccuScan VersaMax240 Activity System and VersaMap programs and used to calculate time spent in each side of the chamber.

#### Forced swim test

We used the standard forced swim test to measure depressive-like behavior. Mice were placed in clear plexiglass cylinders containing 23°C water for 6 min. Video was recorded using EthoVision XT 7 and immobility during the last 4 min was hand-scored by the experimenter using Ethovision’s The Observer XT 10.

#### Acoustic startle response

Mice were assessed for basal behavioral reactivity by testing their startle response to an auditory stimulus, as previously described (Brunssen et al., 2013) using a San Diego Instruments SR-Lab system. Mice were placed into restraint tubes and given 5 min to habituate to the tubes and background noise of 70 dB, followed by six trials of a 40 ms auditory cue of 120 dB, separated by 80 ms. Startle amplitude was collected across a 65 ms sampling window for each trial.

#### Contextual and cued fear conditioning and memory

Mice were assessed for learning and memory of conditioned fear, as previously described (Huang et al., 2013), using the Near-Infrared image tracking system (MED Associates, Burlington, VT, USA). On the first day of this three-day test, mice were placed in a sound-attenuating box and allowed to explore for 2 min before three exposures to a 30-s tone of 90 dB (conditioned stimulus; CS), followed by a two-sec scrambled foot shock of 0.6 mA (unconditioned stimulus; US) separated by a random interval. Freezing behavior during the tone was quantified by the Near-Infrared image tracking system. On the second test day, mice were placed back in the original test chamber and freezing to the context in the absence of the tone presentation was assessed for 5 min. On the third test day, the chambers were modified using a novel odor and inserts to change the surface of the chamber’s walls and floors. After 2 min of habituation, the mice were presented with the CS for 3 min to assess freezing to the cue.

#### Drinking in the dark procedure

We used the standard 4-day DID, a well-established animal model of human binge drinking that generates high blood ethanol concentrations (BECs; ≥80 mg/dl) and has been used to characterize neuromodulators of binge-like ethanol consumption (Sparta et al., 2008; Lowery-Gionta et al., 2012; Sparrow et al., 2012). Three hours into the dark cycle, home cage water bottles were replaced with bottles containing a 20% (*v*/*v*) ethanol solution for 2 h on Days 1–3 and 4 h on Day 4 (binge test day). Tail blood was collected immediately following ethanol access on Day 4 to evaluate BECs.

#### Sucrose and ethanol preference tests

We conducted a sucrose preference test to assess whether deletion of Y2R from GABA neurons induced anhedonia in mice. At the beginning of the dark cycle, mice were presented with two bottles, one containing water and the other containing a 1% (*w*/*v*) sucrose solution, for 24 h. Volume of each bottle’s contents was used to evaluate the degree to which mice preferred sucrose to water. We also conducted a 24-h ethanol preference test in the same manner, except that 20% ethanol (*v*/*v*) was used instead of sucrose and the two-bottle choice began 3 h into the dark cycle.

### STATISTICAL ANALYSIS

Appropriate statistical analyses were used to evaluate the effects of sex and Y2R deletion from GABA neurons, including two-way (sex x genotype) ANOVAs. *Post hoc* *t*-tests with Bonferroni corrections for multiple comparisons to protect against Type 1 error were conducted to make direct comparisons between groups when ANOVAs revealed significant interactions.

## RESULTS

### ELECTROPHYSIOLOGY AND FLUORESCENCE IMMUNOHISTOCHEMISTRY

**FIGURE 2:**
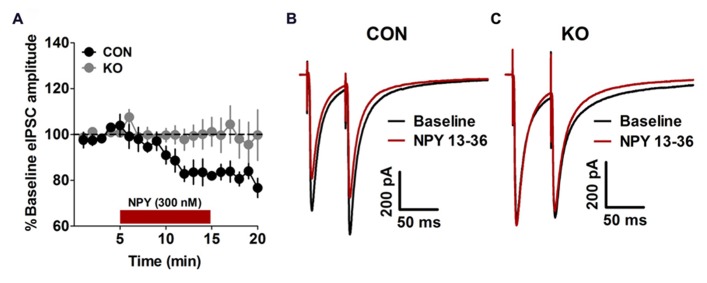
**Inhibitory synaptic transmission. (A)** Bath application of NPY (300 nM) decreased the amplitude of electrically evoked IPSCs in control mice (*n* = 8) but not KO (*n* = 13) mice. **(B,C)** Representative traces of eIPSCs from control **(B)** and KO **(C)** mice before and after bath application of NPY.

**FIGURE 3:**
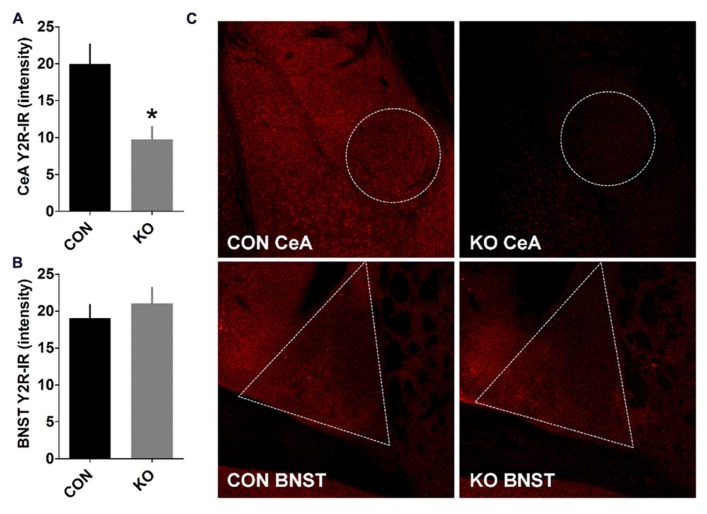
**Y2R-immunoreactivity in the CeA and BNST. (A,B)** Y2R-IR is decreased in the CeA **(A)** but not BNST **(B)** of KO mice compared to controls (*p < 0.05, *n*’s = 4 per group). **(C)** Representative images taken with a 10× objective of Y2R-IR in the CeA and BNST in control and KO mice, with a border highlighting the quantified regions.

We performed analyses of NPY and Y2R content and function to evaluate the effects of deletion of Y2R from GABA neurons on the function of extended amygdala brain regions critical for anxiety, depression, alcohol drinking, and fear behaviors studied here. We found that in the BNST, the ability of NPY to decrease evoked GABAergic transmission, which we have previously demonstrated occurs via Y2R (Kash and Winder, 2006; Pleil et al., 2012), was present in control Y2R-floxed mice [**Figures 2A,B**; *t*(7) = 9.01, *p* < 0.0001] but completely absent in KO mice [**Figures 2A,C**; *p* > 0.55], producing a significant difference between control and KO mice [*t*(12) = 2.97, *p* = 0.012]. Interestingly, Y2R-IR was decreased in the CeA in KO mice compared to controls [**Figures 3A,B**; *t*(6) = 3.17, *p* = 0.019] but not altered in the BNST (**Figures 3A,C**; *p* > 0.50), even though Y2R function was ablated in KO mice (**Figure 2**).

### BEHAVIOR

#### Elevated plus maze

**FIGURE 4:**
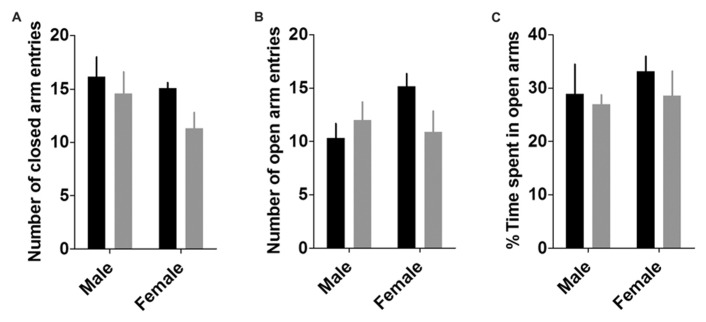
**Elevated plus maze.** There were no effects of sex or genotype on any measures of locomotion or anxiety in the elevated plus maze, including number of closed arm entries **(A)**, number of open arm entries **(B)**, and the percent time spent in the open arms (**C**; *n*’s: CON male = 6, KO male = 6, CON female = 11, KO female = 10).

Two-way ANOVAs revealed that neither sex, genotype, nor their interaction affected any measure of anxiety or locomotion in the EPM (**Figure 4**), including entries to open arms, closed arms, and total arms (*p*’s > 0.05), or percent time spent in open arms (*p*’s > 0.35).

#### Open field test

**FIGURE 5:**
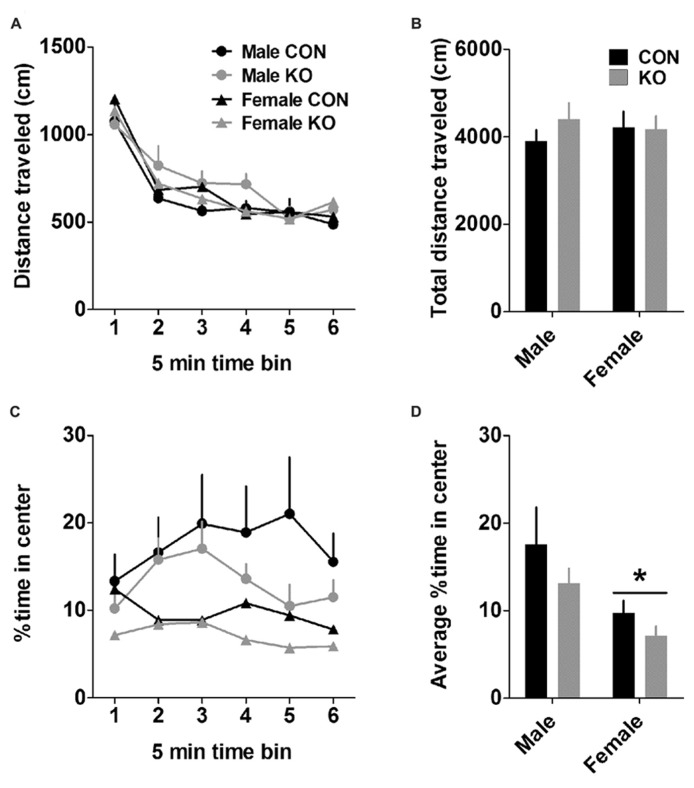
**Open field test. (A)** Distance traveled across 5-min time bins in the open field. **(B)** There were no effects of sex or genotype on the total distance traveled in the open field. **(C)** Percent of time spent in the center of the open field across 5-min time bins. **(D)** Females spent less time in the center of the open field than males (*p < 0.05), suggesting that females display higher basal anxiety than males but do not differ in basal locomotion (*n*’s: CON male = 11, KO male = 10, CON female = 11, KO female = 10).

In the open field test, females displayed more anxiety-like behavior than males (**Figure 5**). While groups did not differ in the total distance traveled during the open field test (**Figures 5A,B**; *p*’s > 0.35), males spent a significantly greater percent of time in the center of the arena than females (**Figures 5C,D**), revealed by a main effect of sex [*F*(1,38) = 7.25, *p* = 0.011)] but no effect of genotype or interaction (*p*’s > 0.10). This sex difference in anxiety-like behavior was recapitulated in the analysis of percent distance traveled in the center of the arena, which also revealed a main effect of sex [*F*(1,38) = 4.67, *p* = 0.037; data not shown] but no other effects (*p*’s > 0.15).

#### Light/dark box

**FIGURE 6:**
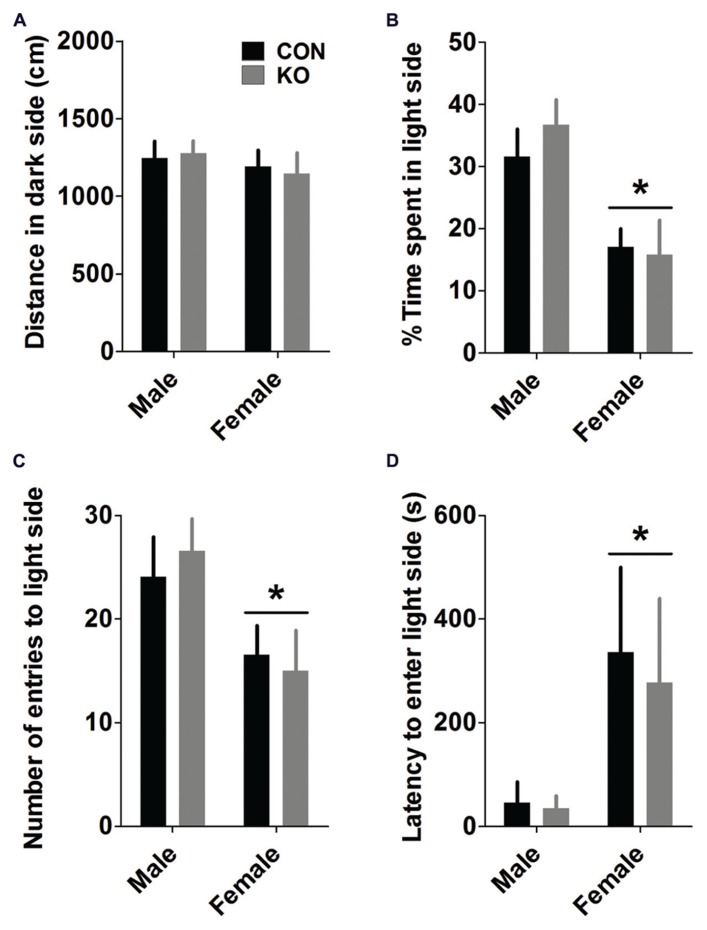
**Light/dark box. (A)** Mice in all groups traveled similar distances in the dark side of the chamber **(A)**. **(B–D)** Females spent less time in the light side of the chamber (**B**; *p’s < 0.001), and made fewer entries into (**C**; *p < 0.001) and had a longer latency to first enter (**D**; *p < 0.05) the light side of the chamber than males, demonstrating greater anxiety-like behavior (*n*’s: CON male = 11, KO male = 10, CON female = 11, KO female = 10).

Similar to the open field test, analyses of light/dark box measures showed that females displayed more anxiety-like behavior than males (**Figure 6**). Males displayed more locomotor activity in the light side [*F*(1,38) = 13.29, *p* = 0.0008] but not the dark side (**Figure 6A**; *p* > 0.35) than females, leading to a greater percentage of their total locomotion [*F*(1,38) = 13.39, *p* = 0.0008] and time in the light side than females [**Figure 6B**; *F*(1,38) = 17.36, *p* = 0.0002]. Males also had a greater number of entries to the light side [**Figure 6C**; *F*(1,38) = 7.71, *p* = 0.009] and a shorter latency to enter the light side for the first time during the test [**Figure 6D**; *F*(1,38) = 5.06, *p* = 0.030] than females, while there were no effects of genotype or interactions between sex and genotype for any of these measures (*p*’s > 0.25).

#### Forced swim test

**FIGURE 7:**
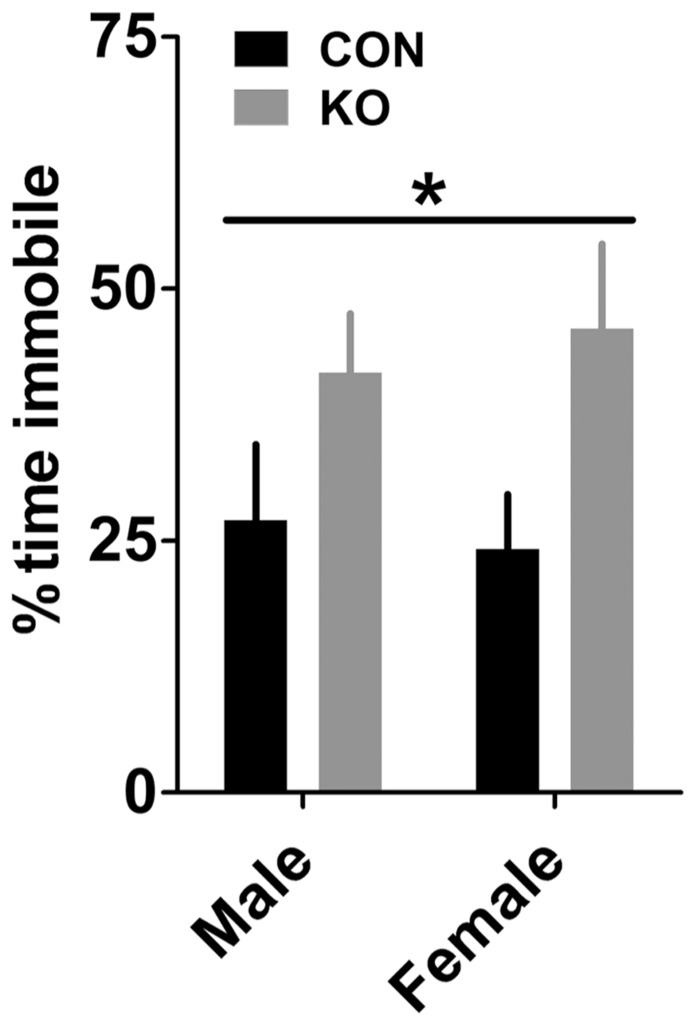
**Forced swim test.** KO mice had increased immobility in the forced swim test compared to control mice (*p < 0.05) but there was no effect of sex, suggesting that deletion of Y2R from GABA neurons increases depressive-like behavior (*n*’s: CON male = 11, KO male = 10, CON female = 11, KO female = 10).

KO mice displayed greater depressive-like behavior in the forced swim test, demonstrated by a larger percent time immobile during the test in KO mice than controls [**Figure 7**; *F*(1,38) = 7.04, *p* = 0.013] but no effect of sex or interaction (*p*’s > 0.60), suggesting that Y2Rs on GABA neurons may play a role in regulating depressive-like behavior.

#### Acoustic startle response

There were no effects of sex, genotype, or their interaction in startle response to the acoustic stimulus (*p*’s > 0.15; data not shown), suggesting that neither sex nor Y2R deletion from GABA neurons affects basal behavioral reactivity.

#### Contextual and cued fear conditioning and memory

**FIGURE 8:**
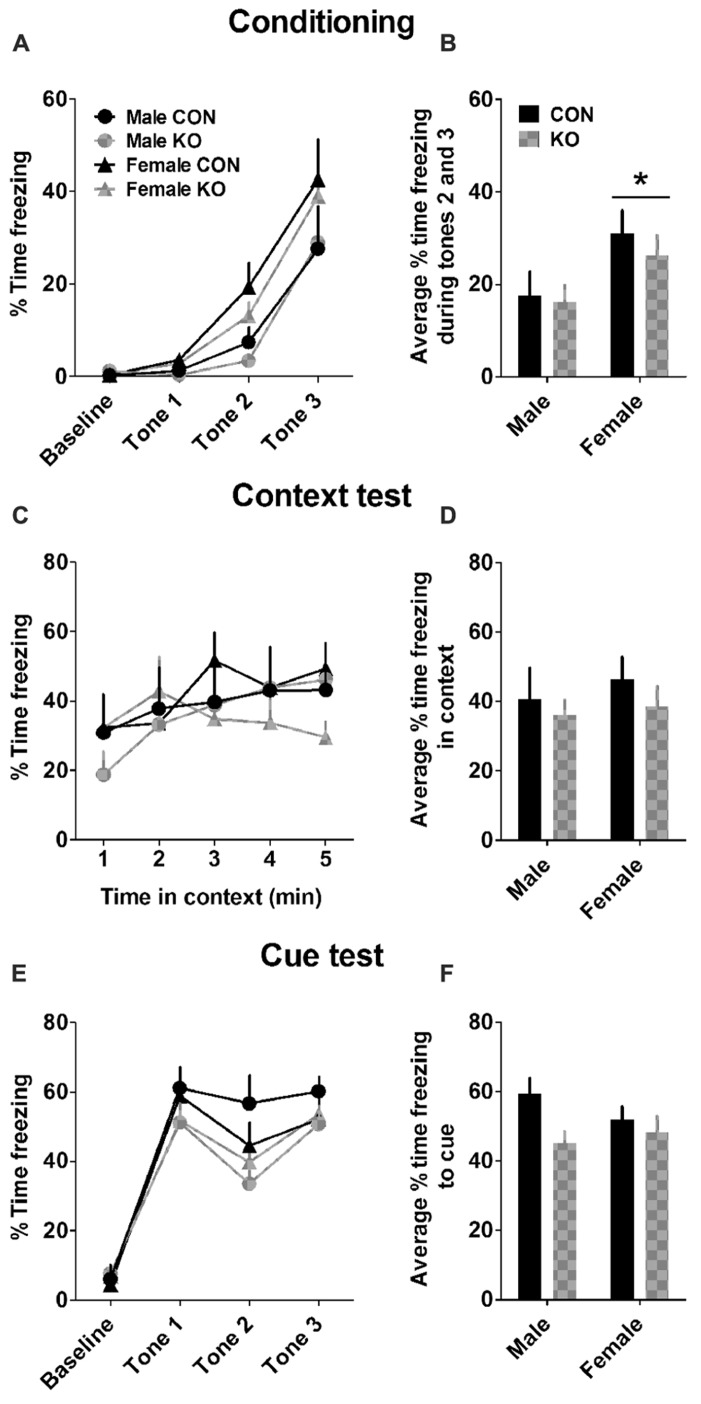
**Contextual and cued fear conditioning and memory. (A)** Percent time freezing during the 2-min baseline and tone presentations during fear conditioning. **(B)** Females froze more during tones 2 and 3 than males during fear conditioning (*p < 0.05). **(C)** Percent time freezing during a 5-min exposure to the same context the day after fear conditioning. **(D)** Neither sex nor genotype altered average percent time freezing during the context test. **(E)** Percent time freezing in a novel context during a 2-min baseline period and three presentations of the conditioning auditory cue. **(F)** KO mice trended toward freezing less to the auditory cue than control mice (*p* = 0.068), while there was no effect of sex (*n*’s: CON male = 5, KO male = 5, CON female = 11, KO female = 10).

Females learned to associate the context/cue to the foot shock more rapidly than males (**Figures 8A,B**), as demonstrated by a main effect of sex [*F*(1,27) = 4.71, *p* = 0.039] but no effect of genotype or interaction between sex and genotype (*p*’s > 0.55) on the average freezing during the second and third shock during conditioning. There were no effects found in the average freezing time during the context test the day after fear conditioning (**Figures 8C,D**; *p*’s > 0.40). On the cue test 1 day later, there was a trend toward a main effect of genotype [**Figures 8E,F**; *F*(1,27) = 3.61, *p* = 0.068] but no other effects (*p*’s > 0.25). These results suggest that females have faster fear learning and that Y2R deletion from GABA neurons modestly impairs cued fear memory but not contextual fear memory.

#### Drinking in the dark procedure

**FIGURE 9:**
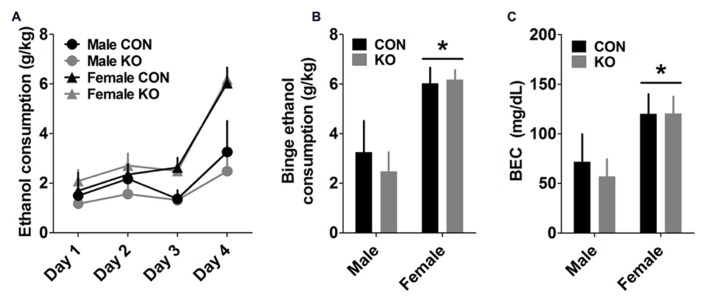
**Drinking in the Dark (DID) binge ethanol drinking test. (A)** Ethanol consumption across the 4-day DID procedure, with 2 h of access to 20% ethanol during the first 3 days and 4 h of access on Day 4 (binge test day). **(B,C)** Females drank significantly more than males on the binge test day **(B)** and had corresponding greater BECs (**C**; *p’s < 0.05; *n*’s: CON male = 4, KO male = 5, CON female = 9, KO female = 9).

Analyses of ethanol consumption across the 4-day DID procedure showed that a sex difference emerged on Day 3 (**Figure 9A**), demonstrated by a main effect of sex [*F*(1,24) = 7.25, *p* = 0.013] but no effect of genotype or interaction (*p*’s > 0.80). This effect persisted on the binge test day (**Figures 9A,B**), shown by a main effect of sex [*F*(1,24) = 19.56, *p* = 0.0002] but no other effects (*p*’s > 0.50). An effect of sex on BEC after the binge ethanol session [**Figure 9C**; *F*(1,25) = 6.40, *p* = 0.018], but no other effects (*p*’s > 0.70), supports this finding.

#### Sucrose and ethanol preference tests

**FIGURE 10:**
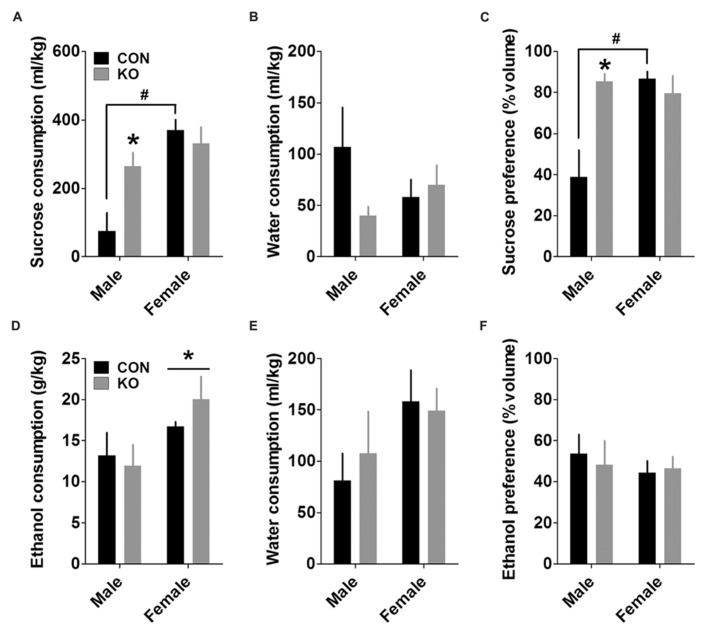
**Sucrose and ethanol preference tests. (A)** Females drank more sucrose than males, and KO males drank more sucrose than control males during a 24-h sucrose preference test (^*^indicates significantly different from control males with *p* < 0.05; ^#^indicates significantly different from control males with *p* < 0.001). **(B)** There were no differences between groups in water consumption during the sucrose preference test. **(C)** Females displayed a greater sucrose preference than males, and KO males had a greater sucrose preference than control males (*indicates significantly different from control males with *p* < 0.01; ^#^indicates significantly different from control males with *p* < 0.001). **(D–F)** Females consumed more ethanol than males during a 24-h ethanol preference test (**D**; *p < 0.05) and trended toward consuming more water (**E**; *p* = 0.069), resulting in no differences in ethanol preference (**F**; *n*’s: CON male = 5, KO male = 5, CON female = 10, KO female = 9).

Analysis of total sucrose consumption during the 24-h sucrose preference test revealed a main effect of sex [*F*(1,25) = 15.89, *p* = 0.0005] and an interaction between sex and genotype [*F*(1,25) = 6.31, *p* = 0.019], but no main effect of genotype (*p* > 0.10). *Post hoc* *t*-tests with Bonferroni corrections showed that control females and KO males drank significantly more sucrose than control males [**Figure 10A**; *t*(13) = 5.14, *p* = 0.0002 and (*t*(8) = 2.87, *p* = 0.021, respectively], while there was no difference between female control and KO groups (*p* > 0.50). A similar analysis on water consumption revealed no effects (**Figure 10B**; *p*’s > 0.05). In addition, analysis of sucrose preference revealed main effects of sex [*F*(1,25) = 7.14, *p* = 0.013] and genotype [*F*(1,25) = 6.24, *p* = 0.019], as well as an interaction between the two [*F*(1,25) = 11.55, *p* = 0.002]. *Post hoc* tests with Bonferroni corrections were also similar to those for sucrose consumption, revealing increased sucrose preference in control females and KO males compared to control males [**Figure 10C**; *t*(13) = 4.68, *p* = 0.0004 and *t*(8) = 3.40, *p* = 0.009, respectively], with no difference between control and KO females (*p* > 0.40). These effects were primarily attributed to very low sucrose consumption and preference in control males.

We performed the same analyses for the ethanol preference test. Similar to results from the DID experiment, females consumed more ethanol than males, demonstrated by a main effect of sex [**Figure 10D**; *F*(1,25) = 6.39, *p* = 0.018] but no other effects (*p*’s > 0.30). There was also a non-significant trend toward increased water intake in females (**Figure 10E**; *p* = 0.069, other *p*’s > 0.55). Together, these consumption levels led to no differences in ethanol preference (**Figure 10F**; *p*’s > 0.45).

## DISCUSSION

In this study, we examined the effects of sex and deletion of the primary NPY receptor, Y2R, from GABA neurons on several measures of anxiety, depression, anhedonia, fear, and ethanol drinking behavior in mice on a C57BL/6J background. We found striking sex differences in these behaviors, with females displaying greater basal anxiety/fear and ethanol consumption in females than males, as observed in the light/dark box, open field test, fear conditioning, DID, and ethanol preference test. Many prior studies in rodents have also found that females display higher levels of basal anxiety, especially those using mice on a C57BL/6J background (Kennett et al., 1986; Johnston and File, 1991; Frye et al., 2000; Frye and Wawrzycki, 2003; Adamec et al., 2006; Dalla and Shors, 2009; Stack et al., 2010; ter Horst et al., 2012b). Notably, heightened stress reactivity, such as anxiety-like behavior on assays used in this study, and faster fear conditioning are two hallmark phenotypes in mouse models of generalized/sustained anxiety (Charney et al., 1993; Cryan and Holmes, 2005; Brinks et al., 2008; ter Horst et al., 2012a,b; Andero et al., 2013; Cacciaglia et al., 2013; Griebel and Holmes, 2013). As women have a higher prevalence of these types of anxiety disorders than men, including generalized anxiety disorder and post-traumatic stress disorder (Kessler et al., 1994, 1995; Gater et al., 1998; Lewinsohn et al., 1998a,b; Bekker and van Mens-Verhulst, 2007), our data are consistent with the current human and mouse literature and suggest that mice on a C57BL/6J background may be good models for sex differences in anxiety in humans.

Interestingly, while we found that females displayed more anxiety-like behavior on the open field test and light/dark box than males, we found no sex differences in the elevated plus maze. While this is a standard assay for anxiety in rodents, there are several previous reports that results in the elevated plus maze were inconsistent with or less sensitive than other tests of anxiety (Johnston and File, 1991; Adamec et al., 2006). Further, evaluation of mouse behavior on a combined apparatus (elevated plus maze, open field test, and light/dark box) showed that mice spent a majority of their time in the elevated plus maze portion of the apparatus, suggesting that it is the least aversive of the three apparatuses (Fraser et al., 2010). Therefore, we and others may not have found a sex difference in anxiety on the elevated plus maze because it is not particularly anxiety-provoking.

As we observed here, other studies have also found greater binge ethanol consumption in female than male mice on a C57BL/6J background (Middaugh and Kelley, 1999; Middaugh et al., 1999; Hall et al., 2003; Rhodes et al., 2007; Agrawal et al., 2013; Melon et al., 2013). Interestingly, there is evidence suggesting that female C57 mice may drink more than males because they are less sensitive to the stimulant and sedative effects of ethanol, independent of BEC (Middaugh et al., 1999). This may be due to modulation of critical circuitry by ovarian hormones, as removal of these sex hormones via ovariectomy has been shown to decrease ethanol consumption in female C57BL/6 mice (Becker et al., 1985). In contrast, in humans, men binge drink more often and in greater excess than women. However, women have smaller gastric ethanol metabolism and less body water volume than men (Marshall et al., 1983; Baraona et al., 2001), leading to higher BECs and greater behavioral effects from the same dose of acute ethanol (Avant, 1990; Frezza et al., 1990; Ammon et al., 1996). As such, women are far more likely to become impaired, suffer greater organ and morphological brain changes, and develop comorbid medical conditions from ethanol consumption (Krasner et al., 1977; Eckardt et al., 1981; Urbano-Marquez et al., 1995; Key et al., 2006; Medina et al., 2008; Squeglia et al., 2012). Therefore, divergent sex differences in ethanol sensitivity in mice and humans may explain why sex differences in ethanol consumption are not consistent between these species.

Surprisingly, we found only one interaction between Y2R deletion and sex, which was observed in the sucrose preference test, a measure of anhedonia. Interestingly, this finding was due to the very low sucrose preference in control (VGAT-ires-Cre -/-, Y2R-floxed +/+) male mice, which may reflect a sex-specific background strain effect on anhedonia, hunger/thirst, or taste, as KO males and both female groups showed high sucrose consumption and preference over water. Given that ethanol and water consumption were not otherwise different between control and KO males, it is most likely that the hedonic value of sucrose is low in Y2R-floxed males. Interestingly, we did not observe any effect of Y2R deletion from GABA neurons on anxiety in either sex. While this was somewhat surprising given the established role of Y2R in these behaviors, we have previously demonstrated that the effects of stress on anxiety-like behavior and on Y2R modulation of GABAergic transmission in the BNST are background strain-dependent (Mozhui et al., 2010; Pleil et al., 2012). These studies demonstrated the stress-resilience of C57BL/6J mice compared to stress-susceptible DBA/2J mice. Similarly, several other studies have found diverging behavioral phenotypes after complete, global Y2R knockout in mice depending on the background of the mice (Redrobe et al., 2003; Tschenett et al., 2003; Carvajal et al., 2006; Zambello et al., 2010). Here, we used mice on a C57BL/6J background because most of our and others’ previous research on alcohol drinking behavior and mechanisms of NPY modulation of circuitry regulating emotional behaviors in mice has used this strain. Specifically, mice on a C57BL/6J strain drink ethanol readily, without the experimenter needing to induce ethanol drinking with a sucrose or MSG fade procedure as other strains require, such as DBA/2J. This allowed us to measure true basal levels of all behaviors in our study, including ethanol drinking. However, we may have observed effects of Y2R deletion from GABA neurons on the behaviors measured had mice been on a more stress-susceptible background, such as DBA/2J. In addition, Y2R-mediated effects on GABAergic transmission and emotional behaviors may emerge after exposure to chronic stress that elicits tonic engagement of the endogenous NPY system.

Our results on the role of Y2R on GABA neurons in depressive-like and anxiety-like behavior are in contrast to previous reports examining site-specific actions of Y2R or manipulation of Y2R in a cell-type non-specific manner. A previous report found that conditional deletion of all Y2R from the CeA decreased depressive-like behavior in the tail suspension test (Tasan et al., 2010). We found that global deletion of Y2R from all GABA neurons increased depressive-like behavior in the forced swim test. Together, these results suggest that (1) the depressive-like effects of Y2R in the CeA are due to modulation of non-GABAergic cell types and (2) Y2R actions on GABA transmission in the CeA, or more broadly throughout the brain, may counteract its actions at non-GABAergic synapses. This is not very surprising given the complex role of Y2R as an autoreceptor and a heteroceptor for many other neurotransmitters implicated in anxiety and depression behaviors. For example, Y2R acts as a heteroceptor on glutamatergic neurons to decrease glutamate release, and it is colocalized with norepinephrine and dopamine terminals (Silva et al., 2001, 2005; Adewale et al., 2007; Nadler et al., 2007; Shoblock et al., 2010; Zambello et al., 2010; Ledri et al., 2011; Stanic et al., 2011). As Y2R is able to decrease the release of both GABA and glutamate, manipulation of one of these functions versus manipulation of both functions would likely result in different behavioral effects. This interpretation is also consistent with our finding that Y2R deletion from GABA neurons did not alter anxiety-like behavior. Several studies have found that complete and global Y2R deletion, systemic Y2R antagonism, or Y2R antagonism in the CeA decrease anxiety-like behavior (Redrobe et al., 2003; Tschenett et al., 2003; Carvajal et al., 2006; Kallupi et al., 2013) or have no effect (Zambello et al., 2010), suggesting that, together with our findings, the anxiogenic effects of Y2R activation in the CeA or more broadly in the brain are due to its role at non-GABAergic terminals.

Although we have previously demonstrated that Y2R modulates GABAergic transmission reliably across neurons in the BNST (Kash and Winder, 2006; Pleil et al., 2012), deletion of Y2R from GABA neurons in this study did not result in less immunofluorescence for Y2R protein in the BNST. However, we functionally confirmed Y2R deletion from GABA neurons in the BNST using slice electrophysiology. As Y2R appears to be ubiquitously expressed throughout the BNST, and deletion of Y2R only from GABA neurons was not be detectable with immunofluorescence, our data suggest that many different types of neurons play host to presynaptic Y2R in the BNST. Future studies combining cell-type and site-specific approaches will help refine our understanding of the loci of Y2R in critical limbic regions and its role in affective and reward-related behaviors.

## AUTHOR CONTRIBUTIONS

Nora M. McCall conducted anxiety-like behavioral assays, maintained breeding colonies, performed immunohistochemical procedures, and helped write the manuscript. Gretchen M. Sprow conducted ethanol and sucrose drinking tests. Eric Delpire generated the Y2R-floxed mice. Todd E. Thiele helped design the study and oversaw ethanol and sucrose drinking tests. Thomas L. Kash designed the study and oversaw anxiety-like behavioral experiments. Kristen E. Pleil helped design the study, performed electrophysiological experiments, analyzed the data, and wrote the manuscript. All authors edited the manuscript.

## Conflict of Interest Statement

The authors declare that the research was conducted in the absence of any commercial or financial relationships that could be construed as a potential conflict of interest.
